# On the Employment of Belladonna in Cases of Frontal Neuralgia

**Published:** 1830-04

**Authors:** J. Claret


### BELLADONNA.

### *On the employment of Belladonna in Cases of Frontal Neuralgia.* By J. Claret.

It is known that the ancients were aware of the stupifying and narcotic action of belladonna, and that they employed it to relieve excessive pain and to procure sleep. Modern practitioners attribute to it the same properties, but use it in a greater variety of maladies. It is now employed to dilate the pupil previous to the operation for cataract; to diminish inflammation of the iris, and those ophthalmies which are kept up by excessive sensibility of the nerves which are distributed to the globe of the eye; as a remedy for hooping cough; and to facilitate the dilatation of the cervix uteri, when its rigidity prevents the issue of the fœtus, or impedes those manual operations which are requisite for its extraction; and lastly, in very diminished doses, as a preservative from measles or scarlet fever * In 1826, Mr. Henry, an English military surgeon, pointed out the efficacy of the extract of belladonna in cases of frontal neuralgia. Since that period, M. Claret has frequently employed it, with the greatest success, in frontal tic douloureux: he considers it, indeed, a specific in such cases. In the course of two years, he has treated five cases with this remedy, and completely cured them. Encouraged by his success, he tried the belladonna in other neuralgic affections; in sciatica, for example, but without any benefit. He attributes the failure of the remedy in such cases to the difficulty of acting upon the deep-seated nerve which is affected. In gastralgia and odontalgia, he has also procured temporary relief from frictions with the belladonna.

The following cases are related to show the dependence that may be placed on the treatment recommended:

Case I. Madame A., æt. twenty-four, of a very nervous temperament, complained of slight pain in the left eyebrow, for which no cause could be assigned. The severity of the pain gradually increased; it became very acute, and extended to the forehead, the vertex, and the globe of the eye: she was also tormented by a sensation of spasmodic contraction in the epigastrium: her pulse was small and frequent; urgent thirst. The attack of pain occurred each morning at about seven o'clock, and continued with more or less violence till four or five in the afternoon: it then

* London Medical and Physical Journal, Jan. 1828, p. 67.

ceased, leaving a feeling of heaviness in the head, and did not again come on until the next day, at the same hour. The occurrence of six regular and uniform paroxysms of pain clearly indicated the case to be one of periodical frontal neuralgia. Ten grains of extract of belladonna, mixed with water so as to render it about the consistence of pomatum, were rubbed in upon the part. The first failed from the imperfect application of the remedy. The second almost instantly diminished the sufferings of the patient, and in three hours the pain was entirely removed. Some weakness of sight was caused by the action of the belladonna upon the retina. The remedy was applied four times in all, and the pain did not return The health of the patient was completely restored, and her vision was unaffected.

Case II. A physician was attacked with the same disease. The pain was at first attributed to irritation of the membrane lining the frontal sinuses, as he had previously laboured under severe catarrh. Fumigations and other local applications were tried in vain. The attacks of pain were periodical and severe. Two frictions effected a cure.

Case III. A woman, æt. thirty-eight, at first complained of a dull pain in the left eyebrow. It quickly became very severe, and extended to the globe of the eye and top of the head, resembling in some degree the clavus hystericus. The pain usually began about ten or eleven o'clock in the morning, and continued until evening, accompanied by so much distress and suffering in the head, that she was unable to attend to any occupation, and was obliged to remain in bed. During the night the pain ceased, and returned the following day nearly at the same hour, continuing its periodic type with great regularity. Eight attacks of this kind had been borne before any medical advice was sought for. Her sufferings at length became insupportable, and she consulted M. Claret. The same remedy was rubbed in on the affected part, and in a few moments the pain disappeared.

Case IV. A poor woman, forty-five years of age, of a very nervous and irritable temperament, had been subject to occasional attacks of headach. A fixed and violent pain on the left eyebrow next came on, and her sufferings were almost insupportable. M. C. found her rolling with agony on her bed, shrieking in a dreadful manner, and unable to rest her head for a single moment. Her pulse was quick, small, and hard; stomach irritable, and would bear no food; extremities cold, and the acute and continued pain caused a tremor over the whole body. She had suffered for four days. The attack commenced about eight o'clock in the morning, subsided towards evening, and returned the next day at the same hour. The belladonna friction was instantly applied. The effect was quick: the pain was soon relieved; and a second friction entirely restored the health of the patient.

Some time afterwards, this woman had another attack, but not so severe as the former. Without any medical advice, she procured the extract of belladonna from a chemist, and cured herself by two applications of it.

Case V. A woman had received a blow on the head, after which she was tormented with severe pain in the eye-brows, forehead, and temples. It was apprehended that some organic mischief had been produced in the brain; but the periodical returns of her sufferings induced M. Claret to consider the symptoms to be merely neuralgic. The extract of belladonna was applied in the same manner, and three frictions restored the patient to health and ease.

In the sixth and last case, the patient was relieved by the same means, after a violent blow on the temple. She afterwards suffered a relapse, and from the symptoms it was evident that the brain had suffered some organic injury.

M. Claret remarks in conclusion, that he considers the extract of belladonna, employed in the above manner, a specific in such cases as those which he has related. His object has been not to speculate as to the *modus agendi* of the remedy, but to state a practical fact of much importance. The treatment recommended by M. Claret is well known, and frequently employed in this country; but we have seen cases of continued suffering from neuralgic affections of the face, and other parts of the body, both in public and private practice, which have resisted various modes of treatment, and in which the external application of the belladonna was not tried. The above satisfactory cases will, no doubt, direct the attention of every practitioner to this mode of treatment.*

* Revue Medicale.

